# From e-service quality to behavioral intention to use e-fitness services post COVID-19 lockdown: When a crisis changes the social mindset

**DOI:** 10.1016/j.heliyon.2024.e30382

**Published:** 2024-04-25

**Authors:** M Rocío Bohórquez, Alejandro Lara-Bocanegra, Rosario Teva, Jerónimo García-Fernández, Moisés Grimaldi-Puyana, Pablo Gálvez-Ruiz

**Affiliations:** a Social Psychology Departament, Faculty of Psychology, University of Seville, Seville, Spain; b Department of Physical Education and Sports, University of Seville, Seville, Spain; c International University of La Rioja, Spain; d Faculty of Law and Social Sciences, Valencian International University, Valencia, Spain

**Keywords:** e-service quality, Consumer satisfaction, Attitudes, Behavioral intentions, Digital platforms, e-fitness service

## Abstract

Lockdowns resulting from the COVID-19 pandemic forced fitness centers to quickly adapt their entire offering to an online format. The subsequent health situation facilitated the maintenance of the online offer and has been a paradigm shift for sports centers. Success in the nowadays situation requires a proper understanding of what factors influence e-service quality and how these factors behave in relation to consumer satisfaction, attitudes toward online fitness services, and behavioral intentions. This research was conducted in April 2020, with 745 participants (492 women, 253 men) completing the Carlson and O'Cass e-service quality evaluation battery. The results showed that e-service quality during the lockdowns predicted attitudes toward the digital platforms and behavioral intentions, and e-service quality predicted attitudes and behavioral intentions. However, attitudes did not predict behavioral intentions; the possible influence of subjective norms and low perceived control in this particular situation is discussed. When offering services on digital platforms, fitness service managers must take into account the importance of the quality of the e-fitness service, but also the social context in which it is offered. Psychosocial functioning in times of crisis influences users' perceived control and their future intention to use online services.

## 1 Introduction

On January 30, 2020, the World Health Organization (WHO) declared that COVID-19 was an international public health emergency; on March 11 of the same year, the WHO upgraded the emergency situation to an international pandemic. This situation caused a prolonged lockdown which, according to Chen et al. [1], increased the risk of sedentary behaviors that contributed to the development of mood disorders such as anxiety and depression. Specifically in Spain, the decrease in the amount and intensity of physical activity practice had more negative impact on women [2].

In this context, fitness centers -as service companies-were forced to adopt strategies that would allow them to adapt and renew themselves as the only way to survive in the short and medium term. Taking advantage of the multiple physical exercise prescriptions provided to the population and in an attempt to adapt to the new circumstances, some of these centers expanded the offer they already had mainly through social media, i.e., Facebook, Twitter, Instagram, YouTube or their own websites [[3], [4], [5], [6]] and others, creating these digital platforms in the face of confining circumstances. In this sense, it was intended to take advantage of the digital channel especially among young people, who increasingly see in Mobile Apps and YouTube channels a valid alternative for physical exercise [7].

According to a report published by Statista [8], the solutions adopted by Spanish gyms during the pandemic were based on offering online classes through their social networks (71.1 %), providing content on their website (68.4 %), using their own mobile application (58.9 %), employing their YouTube channel (44.7 %) and conducting live sessions through video conferencing (40.8 %). This same entity [9] pointed out that in 2021, 90.5 % of fitness centers were still offering online activities after the pandemic. Statistics estimate that a third of the users of fitness centers had not returned to them in 2021, and that the sector has not yet recovered 9 % of the members it had in 2019 [10].

Thus, the objective of this study was to investigate the relationship between the fitness digital platforms offered during lockdown due to COVID-19 and the intention to use them after the periods of confinement. This analysis will allow us to understand the changes that have occurred in the fitness sector since March 2020.

## 2 Theoretical framework

### 2.1 Carlson & O'Cass conceptual framework: from E-service quality to behavioural intentions

To elucidate the intricate relationship between e-service quality and usage/purchase intentions on sports websites, Carlson and O'Cass [11] proposed a theoretical model wherein consumer satisfaction and attitudes toward the use of digital platforms moderated the relationship. The relationship between the e-service quality perceived by the consumer, the satisfaction he/she shows with the service, the attitudes he/she shows about the organization and the behavioral intention toward the products offered has been demonstrated in the literature [12] and specifically, in the sports context [11,[13], [14], [15], [16]].

E-service quality is understood as the degree to which a service is capable of meeting and satisfying the needs of consumers through electronic devices, in which the consumer interacts only with an interface [14]. E-service quality is an antecedent of consumer attitudes about digital platforms [17], whether sports content [11,13,14] or e-commerce [18]. Starting from the consideration of attitudes as learned predispositions to respond to an object or stimulus in a consistently favorable or unfavorable manner [19,20], the importance of different attributes of service quality as a determinant of these toward digital services has been postulated. Different studies have pointed out the attributes of e-service quality: Chen and Wells [17] and Chen et al. [21] pointed out the importance of entertainment, informativeness, and organization. Vijayasarathy [22] highlighted the importance of usefulness and perceived ease-of-use. Carlson and O'Cass [11] identified that perceived usefulness, ease-of-use, entertainment, and a complimentary relationship predicted a positive attitude toward websites.

E-service quality is a determinant of competitive advantage and firms’ long-term success [23] given its relationship with, among other factors, consumer satisfaction [24] in contexts such as sports team websites [11] and in the purchase of Apps [25]. In the case of the sports sector, the e-service quality of sports websites was positively associated with web satisfaction, while shoppers' satisfaction with sports commerce websites strengthened subjects' well-being [26].

Consumer satisfaction, on the other hand, has been defined as the set of affective reactions -of varying intensity- and limited duration about specific aspects of product acquisition and/or consumption [27], for example, online services [28]. Thus, consumer satisfaction with digital services will derive from the overall experience of consumer interaction with the website and its features (for example, ease-of-use) [10,29]. In this sense, consumer satisfaction can be underpinned by functional aspects of durability and safety, as well as others related to enjoyment, knowledge, novelty, and image [30]. However, e-service companies fail to efficiently convert improved safety, awareness, and novelty into consumer satisfaction [30].

The literature has related e-services quality to different behavioral intentions such as the likelihood of revisiting a website [12,14,28], the intention to purchase online and/or via an App [11,25,28,31], recommending acquaintances to visit websites [11,28], or minimizing the probability of access to competing websites [11], achieving greater online competitiveness and establishing itself as an e-commerce advantage [28]. In these quality assessments, consumers evaluate different dimensions related to the e-services received to form an overall assessment of e-service quality, which in turn influences behavioral intentions [13].

Consumer satisfaction with the attributes of a digital platforms (such as response time or ease of navigation) is associated with the formation of positive attitudes toward that digital platforms and the confirmation -or not-of prepurchase expectations [17,21,25]. From the prism of different sectors, in the hotel sector the quality of the digital platforms service contributes to customer satisfaction and to their subsequent engagement and loyalty toward the brand [32]. This relationship was confirmed for sports team websites [11], or online commerce websites [18], and e-commerce Apps [25]. Customer satisfaction, in addition to having a direct influence on attitudes toward a given digital platforms, plays a mediating role between these and e-service quality [11,13].

Consumer satisfaction with digital platforms has been shown to influence behavioral intentions to use websites for sports team content [11] or online sales [25]. Specifically, satisfaction with the use of a digital platforms means that purchases on that website are more likely [18], information about the service is provided to acquaintances [18] or the website is revisited [11,33]. Customer satisfaction, in addition to having a direct influence on behavioral intentions, plays a mediating role between these and e-service quality [11,13].

Attitudes have a direct bearing on consumers' behavioral intentions [20,34]. However, attitudes are not the only predictor of behavior, people's socio-cultural context (the subjective norm) and perceived control also explain the intention to engage in behaviors as outlined in the Theory of Planned Action [35,36]. Recent meta-analyses have revealed that, in fact, subjective norms and perceived control act as moderating variables in the relationship between attitudes and behavioral intentions [37].

In the digital context, the attitude that users of digital platforms have toward them will influence their behavioral intention either positively or negatively [11,17,22,25]. A positive attitude toward a particular digital service will be related to the intention to revisit the digital platforms and to transact on it [22].

### 2.2 E-fitness services in times of crisis

The main adaptation strategy of fitness centers during the lockdown established as a result of COVID-19 and their consequent closure, as well as in the subsequent periods of mobility restrictions and limited capacity of sports spaces, was to make online classes available to their clients and to try to stay connected with them through virtual means [[38], [39], [40], [41], [42]].

Hollasch et al. [43] indicates that traditional gyms were ahead of the curve in using new marketing channels to offer live-streamed classes and online challenges, as well as in employing social media to boost engagement, postings, and visits to mitigate revenue declines and retain their customers. Along the same lines, IHRSA [44], on its China site, indicated that fitness centers' operations staff had made an effort to maintain contact with members through chat groups, encouraging participation through this medium and incentivizing follow through on posed daily routines (and their recording) with rewards when they returned to their fitness center. Webs, WhatsApp Web, and Zoom are the digital platforms that were most frequently used by physical education professionals to guide physical activity during the pandemic [45,46], although other digital platforms have been used in Spain, many of them related to social networks [2].

Following this line, Romero [47] points out that fitness centers have offered free online classes to the entire population, which has meant reconverting and offering virtual services so that their clients could carry out their classes, with their usual sports technician, from their homes. In addition, the willingness shown by specialized companies to give away for a limited time this type of services [48], added to the forced situation of the initial lockdown, resulted in a large number of fitness centers having a high presence in digital platforms through online activities. Romero [47] and González-Serrano et al. [49] points out a digital reconversion of the sector in the face of a paradigm shift. In the USA, online e-fitness courses, classes, and subscriptions were incorporated as the third fastest-growing trend [50]. This technological shift is likely to continue after the pandemic as the benefits and risks of virtual classes are increasingly better understood and work can be done on managing user-service interaction [51]. Along these lines, according to a report by Glofox [52], the fitness centers of the future will have to adapt to this new situation and combine the offer of face-to-face activities with the offer of virtual services (classes, training sessions, …).

According to a survey conducted during the pandemic in the USA [53], 74 % of respondents used a mobile application during the quarantine. Moreover, in the first two quarters of 2020, at the beginning of the pandemic, downloads of applications for physical activity at home grew by 46 % worldwide. In addition, several operators of fitness equipment and material have offered significant discounts on the purchase of products to facilitate the practice of physical activity at home, and the national public television offers in its television schedule content oriented to physical activity, which will have continuity as happens with content for educational purposes (e.g., programs for language learning) or entertainment (e.g., programs aimed at children).

**Fig. 1:**
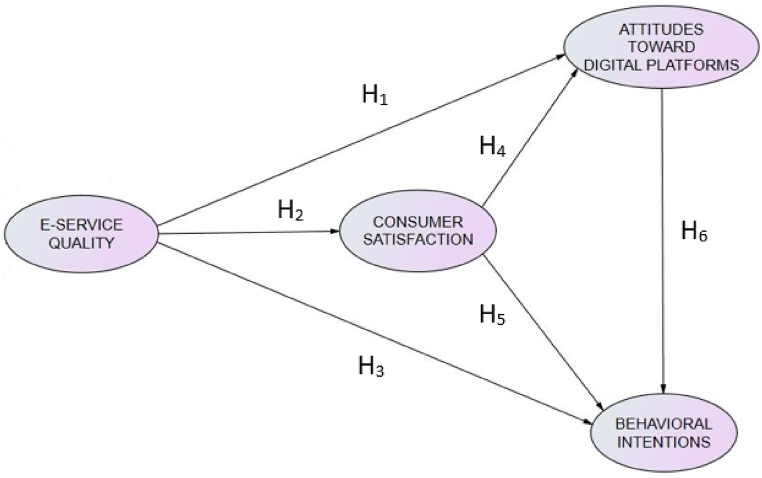
Structural framework.

Consequently, this research means to investigate the relationships established between the e-service quality, consumer satisfaction, attitudes toward digital platforms and the behavioral intentions offered online by fitness centers during the end of the COVID-19 pandemic in Spain. Based on this objective, the following hypotheses were proposed (Fig. 1):

- H_1_. The e-fitness service quality will predict the attitudes toward the digital platforms.
- H_2_. The e-fitness service quality will predict consumer satisfaction.
- H_3_. The e-fitness service quality will predict future behavioral intentions.
- H_4_. Consumer satisfaction will predict the attitudes toward the digital platforms.
- H_5_. Consumer satisfaction will predict future behavioral intentions.
- H_6_. The attitudes toward the digital platforms will predict future behavioral intentions.

## 3 Materials and methods

### 3.1 Participants

The target population of this study was consumers of fitness centers in Spain. A non-probabilistic cross-sectional convenience study was carried out for data collection. Specifically, 780 people participated in the study, of whom three were eliminated from the sample for not residing in Spain, and 34 for giving a biased response (they always answered all the questions with the same value). The final sample included 745 participants, 492 women (66.0 %) and 253 men (34.0 %). In terms of age, similar percentages were obtained in three ranges, between 23.9 % and 25.0 %. A high percentage of the participants lived with their partner during lockdown (73.8 %), of whom 54.6 % have no children. With regards to academic achievement, almost half indicated having a college education (43.2 %) and 81.9 % indicated having an average economic level. Regarding online activities, 69.0 % expressed that they already had such services available before COVID-19, and 64.4 % stated that the fitness center had implemented a digital platforms for training during lockdown. In terms of housing typology, 38.1 % indicated having between 75 and 100 square meters of housing and 39.5 % specified that their housing exceeded 100 square meters, with 71.5 % of the participants having an outdoor area, specifically balcony/terrace (46.4 %) or garden (25.1 %) (Table 1).

**Table 1:** Profile of participants.

Characteristics	Number of respondents	Percentage
*Gender*		
Woman	492	66.0
Man	253	34.0
*Age (years)*		
From 18 to 20	45	6.0
From 21 to 30	178	23.9
From 31 to 40	182	24.4
From 41 to 50	186	25.0
From 51 to 60	120	16.1
More than 61	34	4.6
*Lives with partner during COVID-19*		
No	195	26.2
Yes	550	73.8
*Have children*		
0	407	54.6
1	104	14.0
2	197	26.4
3 or more	37	5.0
*Academic achievement*		
Elementary education	46	6.2
High school	199	26.7
College	322	43.2
Master or Doctorate	178	23.9
*Socioeconomic level*		
High	103	13.8
Medium	610	81.9
Low	32	4.3
*On-line services offered*		
No	26	3.5
Yes, before COVID-19	514	69.0
Yes, during de COVID-19	123	16.5
I don't know	82	11.0
*Training App availability*		
No	46	6.2
Yes, before COVID-19	150	20.1
Yes, during de COVID-19	480	64.4
I don't know	69	9.3
*Square meters of housing (approx.)*		
Less than 75 m^2^	167	22.4
Between 75 and 100 m^2^	284	38.1
More than 100 m^2^	294	39.5
*Availability of outdoor area*		
No	212	28.5
Terrace or balcony	346	46.4
Garden	187	25.1

### 3.2 Instruments

To measure the role of digital platforms services in fitness centers, a questionnaire initially developed by Carlson and O'Cass [11] with 48 items was used. It was based on multi-item scales validated in the context of content-driven e-service web sites, and includes four variables: e-service quality (composed of a total of thirty-four items and four key constructs, specifically usefulness, ease-of-use, entertainment, and complementary relationship) [54], customer satisfaction (a four-item scale) [55], attitudes toward the digital platforms (a five-item scale) [17,56,57], and behavioral intentions (a five-item scale) [58]. Responses were based on a seven-point Likert-type scale ranging from 1 (strongly disagree) to 7 (strongly agree).

The questionnaire was translated into Spanish. To do so, a panel of three bilingual experts with more than five years of experience in sports management used collaborative back-translation techniques to translate the instrument into Spanish [59]. Next, two individuals with competence in both English and Spanish performed a back-translation of the Spanish items to English to maximize the content equivalence and to reduce interpretation difficulties, and the back-translation was the same as the original items and only required minor editing to obtain a final Spanish scale. Then, to determine the content validity we used a validation group composed of four managers of fitness centers and one consultant specialized in the fitness industry.

To find out the physical activity habits prior to and during lockdown, the participants were asked: does your fitness center offer activities through digital platforms? does your fitness center have a fitness App? approximate habitable square meters of the house in which you are confined; does your home have a garden, terrace or exit to outside?

### 3.3 Procedure

A double strategy was organized for data collection. Firstly, we contacted fitness centers to ask about disseminating the link of the on-line questionnaire to facilitate personal contact with their costumers; secondly, we published information regarding the study on various several specific forums related with fitness and sport. The study complied with all ethical research criteria, as well as the voluntary nature of participation, and the anonymity and confidentiality of the participants was guaranteed by requesting their participation in the study and obtaining the informed consent of all the participants.

The questionnaires took approximately 10–12 min to complete. The data was collected over fourteen days, specifically from March 22 to May 5, 2020, coinciding with the period of lockdown due to COVID-19 in Spain. The data were transferred to a spreadsheet to allow a preliminary analysis of them.

### 3.4 Data analysis

Since the original instrument by Carlson and O'Cass [11] evaluated the websites of professional teams and retail sites, for the present work it was necessary to carry out an exploratory factor analysis (EFA) and a confirmatory factor analysis (CFA), thus verifying the factor structure with respect to the original instrument and validating the resulting structure. These phases were necessary to guarantee the validity of the instrument applied to another context, such as digital e-fitness service platforms. The EFA was employed to check the factorial structure using principal components extraction and Oblimin oblique rotation. We had previously tested the factorization conditions using the Bartlett and Kaiser-Meyer-Olkin (KMO) test. The CFA was estimated to confirm the factor structure of the measurement model, using a maximum likelihood (ML) estimator method. Finally, the structural equation model (specifically CB-SEM to test and confirm the theory [60]) was conducted to test the hypotheses and empirically validate the structural model. The adjustment was assessed through following common practice by examining various indices: χ^2^ and its differences of degrees of freedom (χ^2^/df ≤ 5) [61], the comparative fit index (CFI ≥ 0.90), the Tucker-Lewis index (TLI ≥ 0.90), the incremental fit index (IFI ≥ 0.90) [62], the parsimony comparative of fit index (PCFI ≥ 0.80) [63] and the root mean square of approximation (RMSEA < 0.08) [64].

Cronbach's alpha (α > 0.70) [65] and composite reliability (CR > 0.70) [66] scores for each factor were examined to measure the instruments' internal consistency, and the average variance extracted score for each factor was assessed to examine convergent validity (AVE > 0.50) [66]. Discriminant validity was also established when the square root of the AVE for each latent construct exceeded the squared correlations between that construct and any other [67]. The Statistical Package for Social Sciences (SPSS) program and Analysis of Moment Structures (AMOS) software, version 21.0 (IBM Corp, Armonk, NY), was used for the statistical analyses.

## 4 Results

### 4.1 Exploratory factor analysis

The analysis of the measurement model yielded a KMO measure of 0.984 with a significant Bartlett's test (χ2 (1128) = 48834,92; *p* < 0.001). The communalities measuring the variance of the variables are systematically above 0.50 per cent and the factor loadings exceed the minimum acceptable value of 0.40 [66]. These results explain 79.80 % of the total variance explained, indicating that the scales had satisfactory factor structures [68], showing sample adequacy and suitability for completing a factorial analysis.

### 4.2 Confirmatory factor analysis

The findings of an initial CFA indicated a poor fit in the measurement model and showed that the model should be adjusted: χ^2^/df = 7122.39/1059 = 6.72, CFI = 0.876, TLI = 0.868, IFI = 0.876, PCFI = 0.822; RMSEA = 0.088 (90 % CI = 0.086, 0.090). For this purpose, the size of the factor loadings is a criterion used to evaluate the reliability of the indicator with the constructs it intends to measure [68]. Thus, after selecting the items whose factor loading was greater than 0.60 [66,69] a new CFA was reconducted. In this way, four items were removed, specifically two of the complementary relationship scale (CR_4: λ = 0.409; CR_6: λ = 0.520) and two of the behavioral intentions scale (BI_1: λ = 0.512; BI_2: λ = 0.469). Additionally, several items showed high modification indexes (MI). Based on these findings and theoretical knowledge, covariances were included between pairs of error of the following items: ease-of-use 4 and 5 (MI = 52.45), entertainment 5 and 6 (MI = 31.79), entertainment 7 and 8 (MI = 78.49), complementary relationship 7 and 8 (MI = 23.77), attitude toward the digital platforms 1 and 5 (MI = 31.85), as well as 2 and 3 (MI = 87.19). The scale constructed with 44 items appeared to fit the data well, obtaining an adequate model fit: χ^2^/df = 4199.78/870 = 4.82, CFI = 0.910, TLI = 0.902, IFI = 0.910, PCFI = 0.837; RMSEA = 0.078 (90 % CI = 0.070–0.083).

### 4.3 Reliability and validity

The main statistics are detailed in Table 2. Items with factor loadings below 0.60 are deleted in the confirmatory factor analysis of the modified model, denoted in Table 2 with an asterisk.

**Table 2:** Mean, standard deviation (SD), factor loading (λ), composite reliability (CR), average variance extracted (AVE) and Cronbach's alpha (α).

Construct and Items	Mean (SD)	λ	CR	AVE	Cronbach's α
*Usefulness (UF)*			0.95	0.62	0.94
1.I can interact with the digital platforms to get information tailored	4.68 (1.89)	0.64			
2.The digital platforms allows me to interact with it for tailored information	4.50 (1.92)	0.67			
3.The information on the digital platforms is effective	5.49 (1.52)	0.92			
4.Information on the digital platforms is what I need to carry out my tasks	5.24 (1.68)	0.88			
5.The digital platforms adequately meets my information needs	5.29 (1.67)	0.91			
6.The digital platforms have interactive features which help accomplish tasks	4.94 (1.80)	0.81			
7.I trust the digital platforms to keep my personal information safe	5.72 (1.49)	0.75			
8.I trust the digital platforms will not misuse personal information	5.76 (1.55)	0.70			
9.I feel that any information communicated is secure	5.40 (1.54)	0.77			
10.Little waiting time between my actions and the site's response	5.03 (1.67)	0.75			
11.The digital platforms loads quickly	5.43 (1.58)	0.77			
*Ease-of-use (EU)*			0.94	0.72	0.94
1.Display pages within the digital platforms are easy to read	5.61 (1.51)	0.85			
2.The text on the digital platforms is easy to read	5.51 (1.54)	0.89			
3.The digital platforms text/label/menu items are easy to understand	5.54 (1.50)	0.92			
4.Learning to operate the digital platforms is easy for me (C1)	5.80 (1.47)	0.81			
5.It would be easy for me to become skillful at using the site (C1)	5.40 (1.60)	0.73			
6.I find the digital platforms easy to use	5.54 (1.57)	0.86			
*Entertainment (ENT)*			0.96	0.74	0.95
1.The digital platforms are visually pleasing	5.41 (1.61)	0.88			
2.The digital platforms look good	5.64 (1.42)	0.88			
3.The digital platforms are visually appealing	5.23 (1.69)	0.89			
4.The digital platforms have innovative features	4.43 (1.83)	0.78			
5.The digital platforms design is innovative (C2)	5.39 (1.63)	0.91			
6.The digital platforms design is creative (C2)	5.44 (1.56)	0.89			
7.I feel happy when I use the digital platforms (C3)	4.82 (1.72)	0.81			
8.I feel cheerful when I use the digital platforms (C3)	4.88 (1.70)	0.83			
9.I feel excited when I use the digital platforms	5.01 (1.67)	0.83			
*Complementary relationship (CR)*			0.89	0.59	0.88
1.The website projects an image consistent with the fitness center's image	5.55 (1.53)	0.84			
2.The website matches my image of the fitness center	5.39 (1.56)	0.92			
3.The website's image matches that of the fitness center	5.46 (1.58)	0.87			
4.The website allows transactions online	3.68 (1.99)	*			
5.All my business can be completed via the digital platforms	4.25 (1.89)	0.61			
6.Most business processes can be completed via the site	3.99 (1.92)	*			
7.Easier to use the digital platforms than phone, fax or mail	4.96 (1.75)	0.68			
8.The digital platforms are easier to use than phoning a rep'tive	5.06 (1.68)	0.62			
*Consumer satisfaction (CS)*			0.98	0.94	0.98
1.I am satisfied with my decision to use my fitness center's digital platforms	5.57 (1.64)	0.98			
2.My choice to use my team's digital platforms was a wise one	5.53 (1.62)	0.98			
3.I think I did the right thing in using my fitness center's digital platforms	5.55 (1.64)	0.98			
4.The fitness center's digital platforms do a good job of satisfying my needs	5.34 (1.58)	0.90			
*Attitudes toward the digital platform (ATWS)*			0.96	0.84	0.96
1.These are a nice digital platforms (C4)	5.53 (1.54)	0.93			
2.My perception of the fitness center's digital platforms is good (C5)	5.43 (1.58)	0.92			
3.These are a good digital platforms (C5)	5.40 (1.59)	0.93			
4.I have a positive attitude toward the fitness center's digital platforms	5.49 (1.54)	0.85			
5.I like my fitness center's digital platforms (C4)	5.33 (1.68)	0.93			
*Behavioral intentions (BI)*			0.93	0.82	0.92
1.I would not consider switching to another related digital platforms	5.07 (1.80)	*			
2.I will purchase merchandise from the digital platforms in the future	3.48 (2.03)	*			
3.I will revisit my fitness center's digital platforms in the future	5.36 (1.76)	0.83			
4.I will say positive things about my fitness center's digital platforms	5.37 (1.70)	0.94			
5.I will recommend the digital platforms to others who seek my advice	5.33 (1.70)	0.93			

Notes: covariances between pairs of items are ordered from C1 to C5; * = deleted items.

All the Cronbach's alpha coefficients were greater than 0.80. The composite reliability for all the constructs was greater than 0.70, ranging from 0.89 to 0.96 according to the recommended thresholds, and the average variance extracted values ranged from 0.61 to 0.94, exceeding the generally recommended cutoff value of 0.50. Finally, discriminant validity can be established if the square root of the average variance extracted value for each construct is greater than its interconstruct correlation. The square roots of the average variance extracted values for all the seven constructs (diagonal in Table 3) were greater than their correlations with other constructs, indicating discriminant validity.

**Table 3:** Discriminant validity for the measurement model according to the Fornell-Larcker criterion.

	UF	EU	ENT	CR	CS	ATWS	BI
UF	**0.77**						
EU	0.58	**0.84**					
ENT	0.59	0.56	**0.86**				
CR	0.57	0.67	0.44	**0.76**			
CS	0.57	0.71	0.72	0.58	**0.96**		
ATWS	0.59	0.55	0.67	0.56	0.67	**0.91**	
BI	0.60	0.65	0.70	0.53	0.80	0.74	**0.90**

Notes: UF = Usefulness; EU = Ease-of-use; ENT = Entertainment; CR = Complementary relationship; CS = Consumer satisfaction; ATWS = Attitudes toward the digital platforms; BI = Behavioral intentions.

### 4.4 Structural equation model

CB-SEM was performed involving the relationships among e-service quality, consumer satisfaction, attitude toward the digital platforms, and behavioral intentions. We checked for possible collinearity issues, regarding it by looking at the variance inflation factor (VIF), obtaining values ranging from 1.934 to 3.205. Being less than 5, this indicated that multicollinearity is not a problem [70]. The findings showed that the structural relationship model had a moderate level of fit: χ^2^/df = 4421.68/881 = 5.04, CFI = 0.918, TLI = 0.911, IFI = 0.918, PCFI = 0.845; RMSEA = 0.079 (90 % CI = 0.077–0.081).

**Fig. 2:**
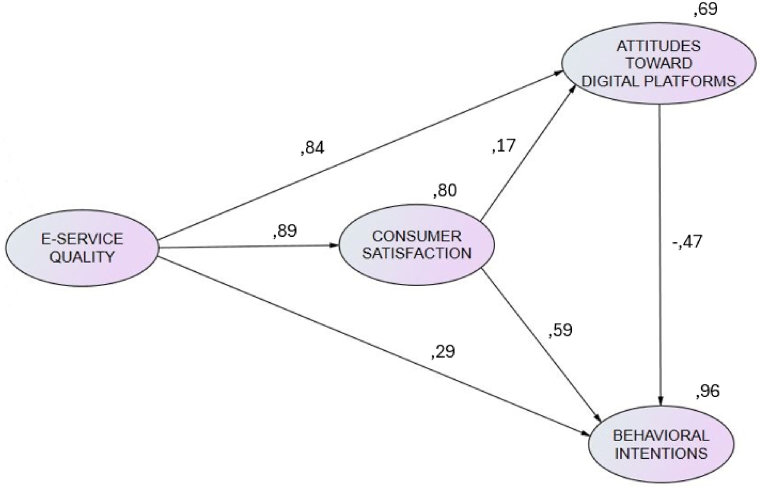
Relationship between constructs with path coefficients (the dotted lines p > 0.05).

The results indicate support for all the causal relationships except ATWS – BI (β = −0.47; *p* = 0.069). In-depth analysis shows that e-service had a significant direct positive effect on consumer satisfaction (β = 0.89; *p* < 0.001) and attitude toward the digital platforms (β = 0.84; *p* < 0.001). It also shows that consumer satisfaction had a significant direct positive effect on attitude toward the website (β = 0.17; *p* < 0.001). In the same way, in relation to behavioral intentions, both consumer satisfaction (β = 0.59; *p* < 0.001) and e-service (β = 0.29; *p* < 0.001) had significant direct positive effects (Fig. 2).

## 5 Discussion

This research proposed that e-service quality would have a positive influence on attitudes toward the digital platforms, and the results confirm this hypothesis [71]. In the electronic world in general, the website design, its reliability, responsiveness, trust, and personalization are determinants of consumer attitudes, so the characteristics of the digital service are determinants of the final success of the business [31]. Thus, our results are consistent with previous findings by different authors for online services related to the sports context [11,13,14,17]. Specifically, the attributes of sports services websites are determinants of e-service quality and, thus, of attitudes toward them [11,17,21,22].

In addition, it was proposed that e-service quality would determine consumer satisfaction with the service. Our results confirmed the hypothesis. These are consistent with those of previous studies analyzing websites of sports teams [11], i.e., the attributes of usefulness, ease-of-use, entertainment and complementary relationship are determinants of e-service quality [11,17,21,22]. Different studies relating fitness service quality to consumer satisfaction in face-to-face contexts already pointed out as determinants of service quality the service convenience [72], the instructor-user relationship and the service's technical competence [73]. It seems that these factors continue to come into play in the relationship between e-service quality and consumer satisfaction in the online context, although the characteristics of the online service must also be taken into account. To our knowledge, this relationship has never before been analyzed in digital sports services (websites, but also other digital platforms), so our results allow us to extend the scientific knowledge needed for sports management.

It was also hypothesized that e-service quality would influence behavioral intentions, an aspect that our results confirmed, allowing us to accept the hypothesis. Knowing that consumers, when evaluating the quality of the service provided digitally, focus on the attributes of the digital platforms interface rather than those strictly related to the service itself [74,75], the App, Web, or Social Network (one digital platform at least) quality used by fitness centers to offer their digital services is important [76]. This quality will determine the probability of employing a website repeatedly [11,14,28], the intention to purchase online and/or in an App [11,25,28,31], recommendations to acquaintances about the online service [11,28], or minimize the probability of accessing competing digital platforms [11], achieving greater online competitiveness and becoming an advantage of e-commerce [28].

On the other hand, it was proposed that consumer satisfaction would have a direct influence on attitudes toward the digital platforms, an influence shown in our results, thus confirming the research hypothesis. Other authors had already found this relationship in the sports sector [11,17,21,25], although they did not test the relationship in digital sports services platforms.

Furthermore, it was expected that consumer satisfaction would determine behavioral intentions: This hypothesis is confirmed given the results obtained. These are consistent with those shown by Carlson and O'Cass [11] for sports team websites, García-Fernández et al. [77] for the use of Fitness Apps, and Choi et al. [78] for online sports practice. However, the relationship, as far as we know, had not been previously tested for fitness digital platforms.

Finally, attitudes toward the digital platforms were expected to determine behavioral intentions. Yet, the results force us to reject this hypothesis since in our study having positive attitudes toward the online sports service did not influence users' intentions to continue employing the service later on. These results contradict those offered by different studies of both face-to-face and digital services [11,17,22,25,77]. However, these results are consistent with those offered, in the same context of crisis, by Alexandris et al. [79], who indicated that users of online e-fitness services had no intention of returning to physical sports centers. Those results could be explained by the moderating effect that subjective norms and social norms have on the relationship between attitudes and behavioral intentions [37] and which we cannot quantify in this study. As Carpí and Breva [80] point out, the Theory of Planned Action is not usually a good framework for predicting those behaviors for which people have a low perception of control. The lockdowns resulting from the pandemic generated important consequences related to the population's perception of low control [81,82], so it is possible to suppose that the perception of uncontrollability prevents attitudes from predicting behavioral intentions.

Thus, the theoretical model of Carlson and O'Cass [11] does not seem to work adequately in crisis contexts in which the subjective norm diverges from the usual one. The main strategy of fitness centers during the COVID-19 lockdown was to adapt their face-to-face services to digital platforms [38], in many cases in an improvised way [47]. As Veiga et al. [46] point out, among the fitness trends in Spain for 2021, exercise Apps for cell phones and online fitness stand out compared to the previous year. This technological change was consolidated in the time after the confinements, but this new service has established itself as one more of the current offers in gyms [49,51,53], so it is important to know what factors will end up influencing the intention to use it, as well as to adapt the offer to the prevailing social vision of these services. These findings imply the necessary adaptation of sports management models in general and online service offerings to social situations. That is, (i) it is necessary to quickly obtain information about the prevailing subjective social norm in each social moment of fitness centers, and (ii) agility in adapting to these changes will ensure the relevance of service offerings in each context.

The applicability of this study lies in the importance and necessity of obtaining relevant information that can be used in times when the population may be subjected to confinement processes and/or inability to leave their homes to practice sports. In this sense, these data can provide great information for the sports sector and serve as a model for those times when action is required.

## 6 Conclusions

In conclusion, this study presents a multidimensional model that explains the relationship between e-fitness service quality during lockdowns due to COVID-19 in March 2020 and behavioral intentions to use such services in the future. The quality of e-fitness services predicts attitudes toward the digital platforms, consumer satisfaction, and behavioral intentions. Similarly, in e-fitness services, consumer satisfaction predicts attitudes toward the digital platforms and behavioral intentions. Despite this, in times of crisis, attitudes toward the digital platforms do not predict future behavioral intentions to use e-fitness services.

The conclusions of this study can be summarized as follows:

- This study presents a multidimensional model that explains the relationship between e-fitness service quality during lockdowns due to COVID-19 in March 2020 and behavioral intentions to use such services in the future.
- The results show us that the quality of e-fitness services predicts attitudes toward the digital platforms, consumer satisfaction, and behavioral intentions.
- In e-fitness services, consumer satisfaction predicts attitudes toward the digital platforms and behavioral intentions.
- In times of crisis, attitudes toward the digital platforms do not predict future behavioral intentions to use e-fitness services.

### 6.1 Limitations and future research

The main limitation of this study lies in not having evaluated the subjective norm about the online practice of physical activity after lockdown or the perceived control over this possibility. This lack of information prevents us from obtaining certainty about the relationship between attitudes toward digital platforms and behavioral intentions in crisis contexts such as the one that occurred during the COVID-19 pandemic. The bibliographical review carried out for the dimensions of Carlson and O'Casśs Model shows an imbalance in the studies found for each dimension: while the literature on the relationship between online service quality and consumption intentions is extensive, other relationships, such as consumer satisfaction and attitudes, have not been as thoroughly examined. Future studies should investigate these moderating factors in the relationship between attitudes and behavioral intentions in the digital context in general and in digital e-fitness services in times of crisis in particular. It would also be necessary to obtain, and compare, our results with those that could be got once home lockdown has been overcome.

Future studies should also analyze the influence of the user inter-face and the sports service itself to determine the weight of each of them in the evaluation of the e-service quality and, thus, improve the overall user experience. In this line, it would also be interesting to analyze the specific characteristics of each type of digital platforms (social network, web, App …) and its influence on e-service quality in the first place and on consumer satisfaction, attitudes toward the digital platforms, and behavioral intentions afterward.

Future studies should re-implement the full model in areas in crisis (e.g., political changes, local epidemics, etc.) to evaluate its feasibility. Likewise, it could also focus on special populations (e.g., pregnant women, sick patients, elderly, etc.) who, for different reasons, have reduced their mobility options and could resort to the virtual service as a way of staying active.

Despite the limitations noted and the questions that remain to be answered, this study provides information that has not yet been addressed about online e-fitness services: the influence of their digital service quality on future intention to use them, as well as the factors that mediate and/or moderate this relationship. Furthermore, knowing how these factors work in situations of home lockdown not only allows us to take advantage of the information in times of a pandemic but also to adapt the offer to user profiles that may occasionally see their ability to use traditional sports services limited, such as people deprived of their liberty, people temporarily displaced from their residence, workers in areas of war, people with medical or psychological disorders who must remain confined to their homes, etc.

## Funding

This research received no external funding.

## Data availability statement

The data used in this article are not available in any repository; however, they will be made available to researchers upon request to the corresponding author.

## CRediT authorship contribution statement

**M Rocío Bohórquez:** Writing – review & editing, Writing – original draft, Visualization, Supervision, Project administration, Methodology, Data curation, Conceptualization. **Alejandro Lara-Bocanegra:** Writing – review & editing, Writing – original draft, Visualization, Validation, Resources, Investigation, Conceptualization. **Rosario Teva:** Resources, Investigation. **Jerónimo García-Fernández:** Writing – review & editing, Supervision, Project administration, Conceptualization. **Moisés Grimaldi-Puyana:** Resources, Investigation. **Pablo Gálvez-Ruiz:** Writing – review & editing, Writing – original draft, Visualization, Validation, Methodology, Formal analysis, Data curation.

## Declaration of competing interest

The authors declare that they have no known competing financial interests or personal relationships that could have appeared to influence the work reported in this paper.
